# Cross-cultural adaptation and reliability of patient asthma knowledge questionnaire in the regional Indian language Marathi: a cross-sectional study

**DOI:** 10.11604/pamj.2024.48.124.37870

**Published:** 2024-07-22

**Authors:** Vishakha Sitaram Nalage, Varoon Chandramohan Jaiswal, Deepika Sohan Burman, Ramya Anand Shetty, Akanksha Amarsingh Chauhan, Amruta Devendra Acharekar

**Affiliations:** 1 Department of Cardiovascular and Respiratory Physiotherapy, Maharashtra Academy of Engineering and Educational Research´s Physiotherapy College, Talegaon Dabhade, Pune, Maharashtra, India

**Keywords:** Bronchial asthma, cross-cultural adaptation, translation, disease-specific knowledge, patient education, Marathi language

## Abstract

**Introduction:**

patient education is the cornerstone of the clinical management of bronchial asthma. The patient asthma knowledge questionnaire is a valid and reliable tool to assess the disease specific knowledge in the patients with bronchial asthma. To the best of our knowledge and literature search, there is no tool available in Marathi language to assess the knowledge of bronchial asthma in patients.

**Methods:**

standard translation guidelines for cross-cultural adaptation were followed. Forward and backward translations were performed by the eligible translators (T1 and T2) as per the guidelines. Synthesis of the translated versions was performed by translators and recording observer (T12). Recommendations by the expert committee were done to develop a pre-final version. The pre-final version was then tested on 30 individuals with bronchial asthma. The reliability of the translated version was assessed by the internal consistency and intra-class correlation coefficient.

**Results:**

thirty (30) bronchial asthma patients were recruited (with a mean age of 63SD±14.36, 16 males and 14 females) to test the pre-final version, and probing of each item was done to test the equivalence. It showed that the patient comprehended the intent behind each inquiry, and that was gauged on the Likert scale. A total of 102 adults (60.8% female and 39.2% male) with a mean age of 41±11 years were included in the study to examine test-retest reliability. Between the total scores obtained from the first and second applications of the questionnaire within a two-week period, there was no discernible variation. The internal consistency reliability (Cronbach´s alpha) was 0.79 and the Intra-class correlation coefficient was 1.000.

**Conclusion:**

Marathi Version of the patient asthma knowledge questionnaire (PAKQ) is cross-culturally adapted and reliable; it will prove to be a beneficial tool to assess the disease-specific knowledge of bronchial asthma.

## Introduction

Bronchial asthma (BA) is a serious public health problem affecting individuals of all age groups and economic conditions. According to the World Health Organization (WHO) in 2016, asthma affected 235 million people globally, with 15 to 20 million of these individuals consisting of the Indian population [1-3]. Bronchial asthma management can be challenging and involves both the individual with BA and their family members. The patients need to learn specifics about their illness to control the disease effectively. It is well documented that insufficient disease-specific knowledge is one of the factors contributing to ineffective control in asthmatics [1-6]. Patient education programs have been found to be useful in increasing disease-specific knowledge and can be a contributing factor in the control of the disease. Developing an individually tailored educational program, and assessment of the disease-specific knowledge is of prime importance [1,5,7]. To successfully customize patient education in bronchial asthma, the disparities between disease-specific knowledge and self-management skills should be evaluated objectively. Self-reported questionnaires assessing patients´ knowledge can be a useful tool in narrowing these disparities, particularly if these tools are validated properly and in patients´ regional language. Specific patient education programs for BA patients use valid and reliable knowledge questionnaires to assess the patient´s knowledge and objectify the effect of the education program [1,5].

Valid and reliable questionnaires are available in various languages including English [1], French [3], Chinese [8], Spanish [9], Portuguese [10], and Turkish [11], but not Marathi. Marathi is the regional language in Maharashtra, India, but the best of our knowledge and literature search, currently there is no method to evaluate the knowledge of Marathi-speaking asthmatic patients. The Patient Asthma Knowledge Questionnaire is designed to explore aspects of asthma care and education program contents, such as asthma-specific knowledge, triggers, diagnostic tests, and asthma management among the patients. Patient asthma knowledge questionnaire was developed and validated by Beaurivage *et al*. in 2017 [1], is a questionnaire on asthma knowledge and self-management that is based on the core requirements outlined in the Global Initiative for Asthma (GINA) strategy report [1]. The measurement properties of the PAKQ were good in terms of content validity, face validity, criterion validity, construct validity consequences validity, reliability, and responsiveness [1]. It's crucial to note that this tool satisfies all of the validity criteria proposed by Pink *et al*. [12]. Hence we wanted to translate an equivalent Marathi version of the patient asthma knowledge questionnaire and check its reliability. The objective of the study is to translate an equivalent Marathi version of a patient asthma knowledge questionnaire and test its reliability. We hypothesised that the Marathi version of patient asthma knowledge questionnaire is well translated and reliable tool.

## Methods

**Study design:** this cross-sectional study was approved by the institutional ethics committee. Permission from the author of the primary version of PAKQ was obtained to translate and adapt the questionnaire in the Marathi language.

**Participants and study size:** sample size of 102 was calculated by using the formula:


$$a+c=\frac{Z^{2}S\left(1-S\right)}{d^{2}}$$

Where: a + c represents the total number of true positives and false negatives. Z is the Z-score, which corresponds to the desired confidence level (here, Z=1.68); S is the sensitivity, which is the proportion of true positives correctly identified by the test (S=0.77) [1]; d is the margin of error or the desired precision (here, d=0.07). Using purposive sampling, diagnosed cases of bronchial asthma of age more than 18 and with their mother tongue Marathi [1,8] who represented range of asthma severities, duration of the asthma, age, and gender were invited to participate. In Table 1 shows the demographic distribution of the participants. Participants were excluded if they had COPD or any chronic lung conditions [1,8]. Mean, median and standard deviation of all the participants is described in Table 2.

**Table 1:** distribution of age and gender of the participants

Valid	Frequency	Percent (%)
Female	62	60.8
Male	40	39.2
Total	102	100.0
Age	41±11 years	
Severity	Mild (n=62)	(60.78%)
	Moderate (n=40)	(39.21%)
Duration	Mean: 11.6 Years	

* Severity classification as per GINA Guidelines 2019

**Table 2:** mean, median and standard deviation of the participants

Number of cases	Score	Total score of 1^st^ test	Total score of 2^nd^ test
Valid	102	102	90
Missing	0	0	12
Mean	47.36	47.36	47.26
Median	48.00	48.00	48.00
Std deviation	3.715	3.715	3.713

**Study settings and data sources:** individuals diagnosed with bronchial asthma according to the GINA guidelines 2019, visiting the pulmonary outpatient department of Dr. Bhausaheb Sardesai Talegaon Rural Hospital were recruited in this study from July 2021 to December 2021.

**Variable:** the patient asthma knowledge questionnaire, is designed to explore aspects of asthma care and education program contents, such as asthma-specific knowledge, triggers, diagnostic tests, and asthma management among the patients. The patient asthma knowledge questionnaire was developed and validated by Beaurivage *et al*. in 2017 [1], is a questionnaire on asthma knowledge and self-management that is based on the core requirements outlined in the Global Initiative for Asthma (GINA) strategy report [1]. The measurement properties of the PAKQ were good in terms of content validity, face validity, criterion validity, construct validity consequences validity, reliability, and responsiveness [1]. It's crucial to note that this tool satisfies all of the validity criteria proposed by Pink *et al*. [12].

**Study procedures:** guidelines prescribed by Beaton *et al*. 2000 [13] to translate and adapt the self-report measures were followed to form the Marathi version of PAKQ. Six steps were completed to translate and adapt the PAKQ in Marathi Version;

**Step 1; forward translation:** the questionnaire was individually translated into Marathi by two bilingual translators with their mother tongue being Marathi (FT1 and FT2 Versions). Forward translator 1 was from medical background and forward translator 2 was from the community with no medical knowledge. Both translators submitted the report along with the translated copy provided to them. Local language, patient and grammatical contexts were considered by the translators during the process.

**Step 2; synthesis of the FT1 and FT2:** a common synthesized version T-12 was developed after reaching a consensus between the two forward translators and the recording observer. The English version of the questionnaire was taken as a reference during the entire process. A documented report of the synthesis process was maintained for further reference.

**Step 3; backward translation:** the synthesized version (T-12) was translated back into English by two back translators individually. Both the back translators were completely blind to the original concept and the questionnaire. Two back translated versions (BT1 & BT2) were submitted by the translators and synthesis was achieved (BT-12). BT-12 version was comparable to the original version of PAKQ. So, no changes were made by the authors in the T12 version.

**Step 4; expert committee:** pre-final version was prepared by the panel of experts consisting of a research methodologist, chest physician, language experts and all the translators. Expert committee recommendations were to include of the Indian brand name drugs and to keep the cognate words as it is in the questionnaire.

**Step 5; test for the pre-final version:** thirty (30) consecutive diagnosed cases of bronchial asthma visiting the pulmonary outpatient with a mean age of 63 (SD= 14.36, 16 males and 14 females) completed the questionnaire and were probed for the exact understanding of the items. All the participants were completed and probed under the supervision of same research assistant. All the participants completely understood the items of the pre-final version and no further changes were required.

**Step 6; submission of the reports:** all the reports and translated versions along with the final Marathi version of PAKQ was submitted to the original developers of the PAKQ and their approval was obtained. Test-retest reliability and internal consistency of PAKQ Marathi version. Informed consent was obtained from all the participants. All the patients filled in the questionnaire twice with a gap of 2 weeks. Test-retest reliability was calculated using the intraclass correlation coefficient (ICC) and Cronbach´s alpha coefficient was used to assess internal consistency.

**Statistical methods:** all the data processing and testing [8] was performed using IBM SPSS software 28.0 Version. Descriptive statistics were used to summarize the demographic characteristics and presented as n (%) or mean, standard deviation. Cronbach´s alpha was used to test internal consistency and test re-test reliability was tested using intra-class correlation.

## Results

**Participants and descriptive data:** a total of 102 adults (60.8% female and 39.2% male) with a mean age of 41±11 years were included in the study. According to the Global Initiative for Asthma (GINA) guidelines (2019), 60.78% of participants were considered with mild asthma, 39.21% with moderate asthma with an average duration of 11.6 years as shown in Table 1. For test-retest reliability, all the participants were recruited in order to complete the questionnaire twice. In the first observation, 102 (n=102) participants completed the questionnaire whereas in the second observation, only 90 (n=90) participants were able to complete it. Hence, we had 12 withdrawals since those participants were unable to make it to the hospital due to distance barriers.

**Outcome data:** in terms of internal consistency, the Marathi version of the PAKQ's Cronbach's alpha was estimated as 0.79, while the intraclass correlation coefficient was examined for test-retest reliability and is 1.000.

**Main results:** the Marathi version of the PAKQ has excellent intra-class reliability, as evidenced by the measurement of the intra-class correlation coefficient whereas the internal consistency was found to be acceptable by evaluating Cronbach´s alpha. This result revealed that all questions are consistent and measure the same concept [8].

## Discussion

The study aimed to translate, adapt, and evaluate the test-retest reliability and internal consistency of the PAKQ in Marathi language. Although there are a number of questionnaires to study the bronchial asthma knowledge this is the first study intended to translate and adapt the questionnaire for Marathi-speaking individuals. The questionnaire was adapted as per the regional context according to the standard guidelines [13]. As per recommendations of the expert panel pharmacological drugs brand names in the original version were changed to the brand names of the Indian version eg: ( Budicort, AB Phylline, Sereflo, Montair, Asthalin, Foracort ) [14]. A complete and good understanding of all the items was seen during the testing of the prefinal version with 30 diagnosed cases of bronchial asthma. The test-retest reliability of the translated and adapted questionnaire was done using the interclass correlation coefficient. The interclass correlation coefficient measured for test-retest reliability was 1.000 with a 95% confidence interval as shown in Table 3 states the good reliability of the questionnaire. Whereas Cronbach´s alpha value calculated was 0.79 showing good internal consistency.

**Table 3:** intraclass correlation coefficient for test-retest reliability

Measures for test-retest reliability	Intraclass correlation	95% confidence interval		F test with true value 0
		Lower bound	Upper bound	df1
Single measures	1.000	1.000	1.000	89
Average measures	1.000	1.000	1.000	89

The original version of the PAKQ has already been tested for validity by the authors of the original version and published elsewhere [1]. The 54-item PAKQ Marathi version is, therefore, a valid and reliable tool with content that aligns with current international asthma guidelines and may therefore be of great help in regional clinical settings [1,3,8]. The questionnaire is easy to administer, appropriate, and intelligible to the target regional population to study the disease-specific knowledge. Marathi language has diverse dialects and a vast geographical regional population speaking the language so there can be few contextual differences while addressing the questionnaire to the entire region which can be addressed by closely monitoring the individual while completing the questionnaire. Also to the best of our knowledge, there is no Marathi asthma knowledge questionnaire previously verified our study did not compare the Marathi PAKQ with standard questionnaires to obtain concurrent validity [8].

The results of this study should be interpreted with caution. First, there may be a selection bias as the participants were recruited from only one tertiary health care center. Second, baseline educational qualification and socioeconomic criteria, which can affect the disease knowledge were not taken into consideration during the time of recruitment. Finally, it is important to validate and test the questionnaire with diverse participants with different cultures, demographics, and educational levels to confirm the generalizability of the tool. To summarize disease-specific knowledge is one important prognostic indicator of bronchial asthma. The lack of disease-specific self-report measures in the regional languages is a major barrier to the assessment of disease-specific knowledge of the individuals and implementation of the specific goal-based structured patient education programs. The questionnaire can be further utilized to study the disease specific knowledge and implementing the various bronchial asthma education programs, which is the integral part of the bronchial asthma management.

## Conclusion

Marathi version of the PAKQ is cross-culturally adapted and reliable; it will prove to be a multi-dimensional tool to assess the disease-specific knowledge of bronchial asthma amongst the Marathi speaking individuals.

### What is known about this topic

- *Patient education for asthma and its assessment using self-report measures is a well-established practice;*
- *Patient asthma knowledge questionnaire is already translated in English and Chinese languages and are both tested for their psychometric properties*.

### What this study adds

- *This study has translated and adapted patient asthma knowledge questionnaire in regional Indian language Marathi;*
- *To the best of our knowledge, there is no instrument available to study the asthma specific knowledge in Marathi speaking individuals;*
- *This questionnaire will enhance the scope of asthma education in the clinical settings of Maharashtra India*.
